# A pilot evaluation of magnetic resonance imaging characteristics seen with solid papillary carcinomas of the breast in 4 patients

**DOI:** 10.1186/s12885-017-3518-8

**Published:** 2017-08-07

**Authors:** Lina Zhang, Ling Zhuang, Chang Shi, Yanwei Miao, Weisheng Zhang, Qingwei Song, Jianyun Kang, Zhijin Lang, Xuegang Xin, Ailian Liu, Jiani Hu

**Affiliations:** 1 Department of Radiology, First Affiliated Hospital of Dalian Medical University, Dalian, 116011 China; 2 Department of Oncology, Wayne State University School of Medicine, Detroit, USA; 3 Department of Pathology, First Affiliated Hospital of Dalian Medical University, Dalian, China; 4 Department of Biomedical Engineering, Southern Medical University, Guangzhou, Guangdong China; 5 Department of Radiology, Wayne State University, Detroit, USA

**Keywords:** Solid papillary carcinoma, Magnetic resonance imaging, Diffusion magnetic resonance imaging, Magnetic resonance spectroscopy

## Abstract

**Background:**

Solid papillary carcinoma (SPC) is a rare variant of breast papillary carcinoma with unique pathological morphology and biological behavior. There is only one case report on T_1_-MRI of SPC. In this study, we report our findings on this new category of papillary carcinoma to fill the gap in MRI characterization of SPC.

**Methods:**

This retrospective study included four pathology-confirmed in situ SPC patients. Conventional MRI, diffusion weighted imaging (DWI), and magnetic resonance spectroscopy (MRS) were performed with a 1.5 T whole-body MR scanner before surgical operation. The following characteristics of each lesion were recorded: signal intensity on T_2_WI/STIR and T_1_FSPGR, morphology, maximum lesion size, and time intensity curve (TIC) on dynamic contrast enhancement MRI (DCE-MRI), apparent diffusion coefficient (ADC) value from DWI, and Cho peak from MRS.

**Results:**

Signal intensities of all lesions were heterogenous on T_2_WI/STIR and T_1_FSPGR. Mass enhancements were observed for all lesions with either oval or irregular shapes on DCE-MRI. The maximum lesion size ranged from 0.8 cm to 3.2 cm. All lesion margins were circumscribed, and internal enhancements were homogeneous or heterogeneous from DCE-MRI. TIC appeared with a rapid increase in initial contrast phases of all lesions. All lesions on DWI (b = 1000s/mm^2^) were slightly hyperintense with an ADC value range of 1.3 × 10^−3^ mm^2^/s to 1.9 × 10^−3^ mm^2^/s. Cho peak was absent at 3.2 ppm for all lesions.

**Conclusions:**

MRI characteristics of SPC include heterogeneous signal intensity within the lesion on T_2_WI/STIR and T_1_FSPGR, mass enhancement with circumscribed margins, either oval or irregular shapes, and a rapid initial enhancement of TIC on DCE-MRI. ADC values and the absence of Cho peak may provide valuable information to distinguish SPC from other invasive breast carcinomas.

## Background

Papillary carcinomas constitute 1–2% of breast carcinomas in women. Solid papillary carcinoma (SPC) is a rare variant of papillary carcinoma with unique pathological morphology and biological behavior [1, 2] and has recently been classified as a new category of breast papillary carcinoma by the World Health Organization (2012), differentiating it from the previous classification as a type of intraductal papillary carcinoma [1, 3–5]. Although still under investigation, the prognosis of SPC seems to be better than that of intraductal papillary carcinoma (the most common papillary tumor) because of SPC’s unique pathological pattern [1–3].

Pathologically, SPC is characterized by round, well-defined nodules composed of densely low-grade ductal cells separated by fibrovascular cores, leading to a morphologically solid growth pattern at low magnification [1, 2]. An underlying fibrovascular stromal network and a solid morphologic appearance are typically observed for SPC. This is in contrast to the papillary fronds covered by stratified columnar cells with uniform hyperchromatic nuclei seen in intraductal papillary carcinoma [1, 3–5]. For Immunohistochemistry (IHC) analysis, neuroendocrine differentiation is commonly presented in SPC, but not in intraductal papillary carcinoma [1, 3].

MRI is widely used to examine breast lesions, including many varieties of papillary tumors [4–8]. However, there is only one case report on T_1_-MRI of SPC [9]. The paucity of SPC imaging research is likely due to the relatively new classification of SPC as a unique papillary tumor. Therefore, this study aims to fill the gap in the current MRI knowledge of SPC by examining this papillary carcinoma using multiple MR image modalities including T_1_W and T_2_W MRI, DCE-MRI, DWI and MRS.

## Methods

### Patient selection

Four SPC patients treated in our center between January 2010 and September 2014 were included in this study. This was a single institution retrospective study approved by the ethics committee of First Affiliated Hospital of Dalian Medical University (Dalian, China). The requirement of written informed consent was aquired. All patients were diagnosed as SPC in situ through pathology after breast conserving therapy (case 2) or modified radical mastectomy (case 1, 3, and 4). Stage of the patients were PT1N0M0 (Case 1 and 3) and PT2N0M0 (Case 2 and 4) indicating no lymph node involvement. It is important to note that one patient (case 3) had a conservation surgery in 2010 due to the presence of a mucinous carcinoma composited with invasive ductal components at a different site within the same breast, while the other three patients had no breast operation history. All patients had a unilateral lesion.

### MR imaging

All MR examinations were performed on a 1.5 T whole-body MR scanner (Signa, Excite, HDx, General Electric Healthcare, Milwaukee, WI) with a dedicated 8-channel breast coil.

The MRI protocols were as follows: 1) conventional MR scan sequences included axial T_1_ fat-saturation spoiled gradient recalled echo (FSPGR), sagittal fat-saturation FSE T_2_WI, and axial short Tau Inversion Recovery (STIR) with the imaging parameters listed in Table 1; 2) Axial dynamic 3D T_1_ FSPGR (Volume Imaging for Breast Assessment, VIBRANT) sequence (total 8 phases, acquisition time = 57 s/one phase) performed after Gadolinium injection given via a catheter placed in the antecubital vein via a power injector at a rate of 2.0 ml/s with a dose of 0.1 mmol/kg followed by 20 ml saline. The first acquisition started 25 s after contrast injection; 3) DWI (b = 1000s/mm^2^) with repetition time/echo time (TR/TE) of 6050 ms/84.3 ms, slice thickness/slice spacing of 5 mm/1 mm, FOV of 30 × 32 cm^2^ and reconstruction matrix size of 256 × 256; and 4) Single voxel point resolved spectroscopy (PRESS) ^1^H–MRS sequence (TR/TE = 1000 ms/144 ms, NEX = 1, reconstruction matrix size = 16 × 16, acquisition time = 260 s).

**Table 1:** Conventional MRI scanning parameters

Sequence	TR (ms)	TE (ms)	TI (ms)	Slice thickness (mm)	Slice spacing (mm)	FOV (cm × cm)	Matrix	Nex
T_1_ FSPGR	3.0	1.2	7	2.1	0	30–32	448 × 350	1
Fat saturation T_2_WI FSE	2860	88.4	/	5	1	20–24	256 × 192	3
STIR	3620	75.7	160	5	1	30–32	288 × 224	1

DWI and ^1^H–MRS were both performed before contrast injection. As ^1^H MRS has become an adjunct to dynamic contrast enhanced MRI (DCE-MRI) in the clinical evaluation of breast lesions [10], MRS is added to our protocol to investigate the performance of MRS in SPC diagnosis.

### Image analysis

All images were transferred to a GE workstation (Advantage Windows 4.5; General Electric, Madison, WI, USA) for image processing and were interpreted by two radiologists with more than five years of diagnostic experience. Consensus was reached on cases in which there was a diagnostic discrepancy between the two readers, which was used in the final MRI analysis. The following lesion characteristics were recorded: 1) signal intensity on T_2_WI/STIR and T_1_ FSPGR, 2) morphology and maximum lesion size on dynamic contrast enhancement MRI (DCE-MRI), 3) time intensity curve (TIC) from DCE-MRI, 4) apparent diffusion coefficient (ADC) value from DWI, 5) Cho peak from MRS.

## Results

**Fig. 1:**
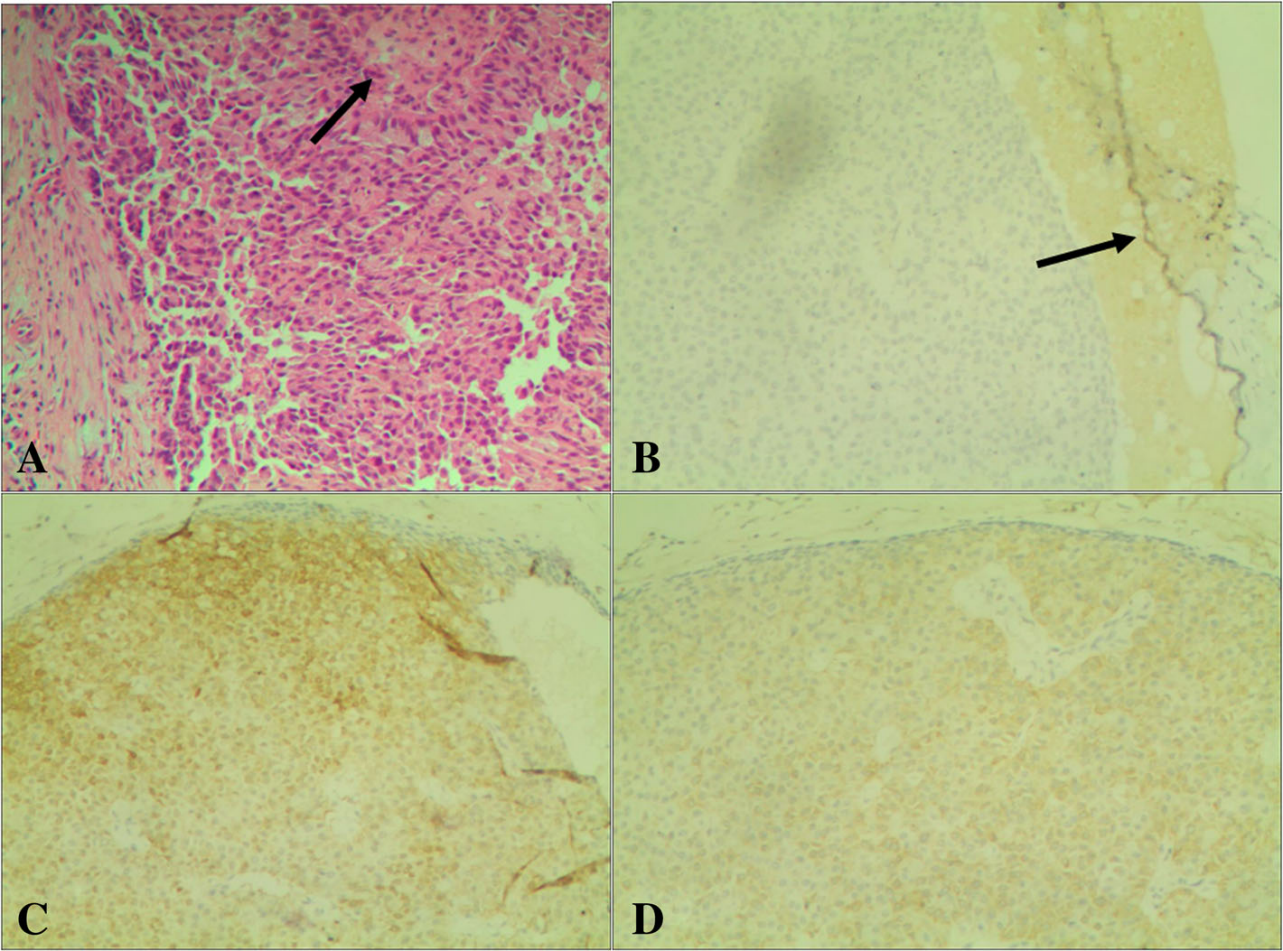
Breast SPC histography and IHC finding. HE shows a duct dilatation with a relatively bland population of epithelial cells with ovoid to elongated nuclei, indistinct nucleoli, and scant mitoses. Fine congested fibrovascular septa course into the solid epithelial islands (*black arrow*) (10 × 20). **a** CD10 shows retention of myoepithelial cells at the periphery of the epithelial islands on IHC (*black arrow*), indicating a non-invasive process. **b** NSE is diffusely expressed in the cytoplasm of the epithelial cells on IHC (*yellow stained*), confirming their neuroendocrine nature **(c).** Syn is diffusely expressed in the cytoplasm of the epithelial cells on IHC (*yellow stained*), confirming their neuroendocrine nature **(d)**

The age of the four patients ranged from 66 years old to 79 years old. Clinical findings including patient age, location, bloody nipple discharge, and duct ectasia are listed in Table 2. For cases 1, 3, and 4, all lesions were located in the medial quadrant of the breast. For case 2, the lesion was located in the lateral quadrant of the breast. For cases 1 and 4, patients had bloody nipple discharge accompanied by duct ectasia. For cases 2 and 3, the patients showed no bloody nipple discharge and had no dilated ducts. Histography and IHC results are shown in Fig. 1 a-d. Case 2 and 4 were associated with a mucinous component on pathology. No lymph node metastasis was found in any case, and all cases showed no evidence of metastasis or recurrence at one-year follow up.

**Table 2:** Clinical findings of SPCs

Case	Age (years)	Location (quadrant)	Bloody nipple discharge	Duct ectasia
1	66	right medial	yes, 20 days	yes
2	70	right lateral	no	no
3	72	right medial	no	no
4	79	left medial	yes, half year	yes

**Fig. 2:**
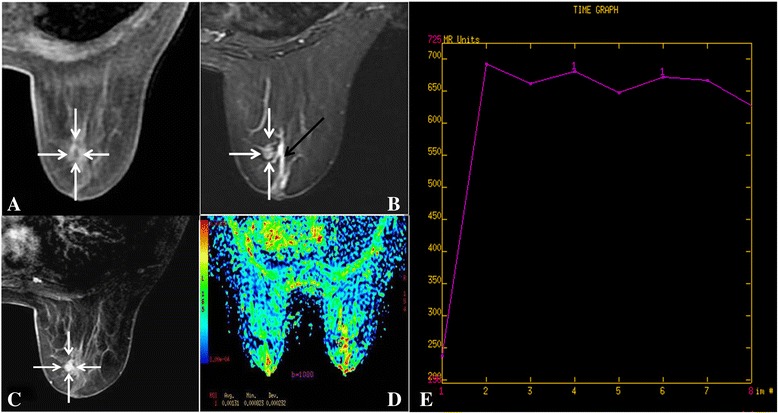
Right Breast SPC (case1). T_1_ FSPGR (pre-contrast) shows a hypo-iso intensity signal mass (*white arrow*). **a** STIR shows an iso-hyper intensity signal mass (0.8 cm, *white arrow*) with adjacent duct expansion (*black arrow*). **b** T_1_ FSPGR (contrast) shows an oval, circumscribed mass with homogeneous enhancement (*white arrow*). **c** ADC map shows ADC = 1.31 × 10^−3^ mm^2^/s (b = 1000s/mm^2^). **d** TIC, rapid increase (initial phases) and plateau type (delayed phases). **e**

**Fig. 3:**
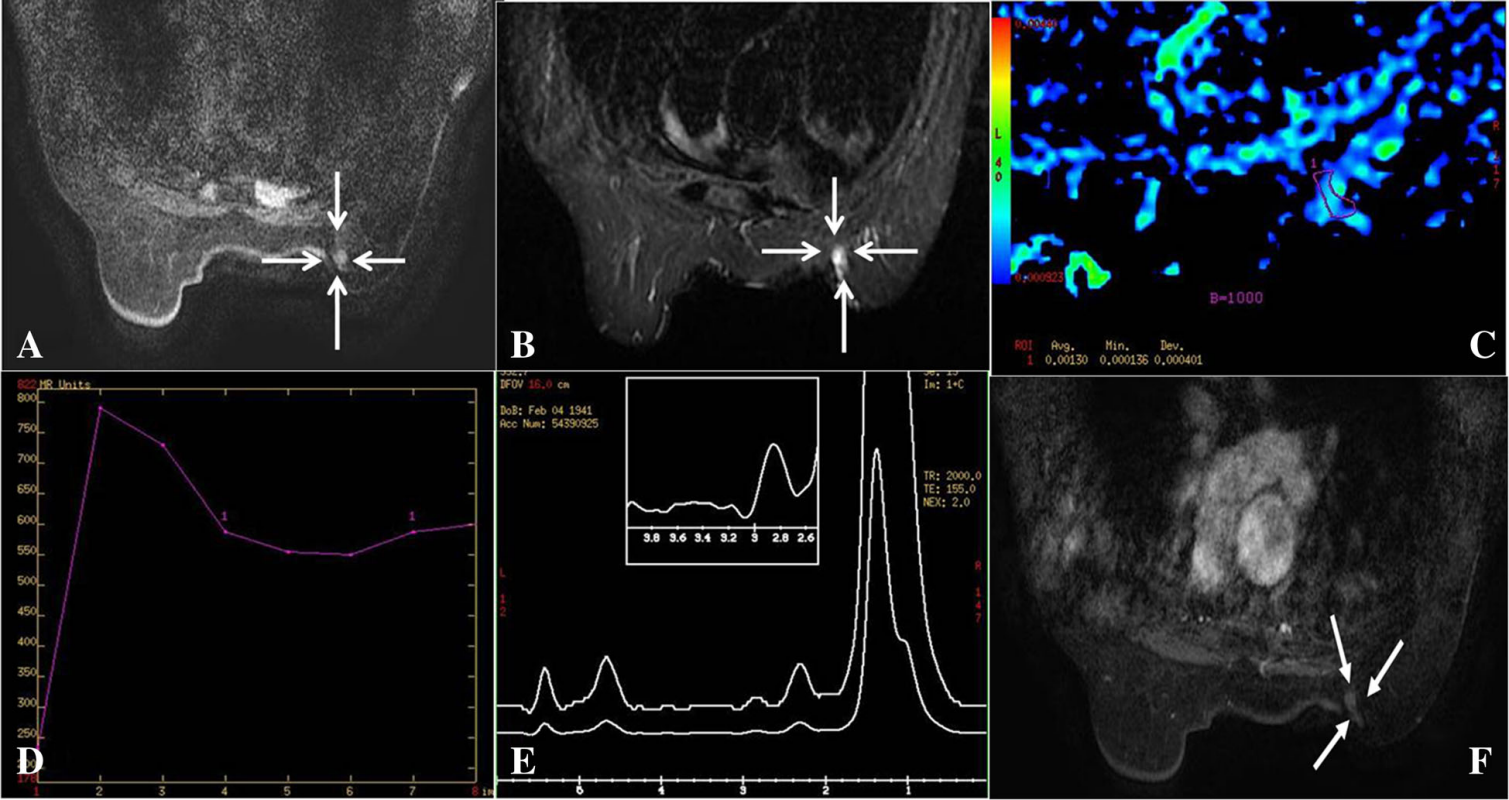
Right Breast SPC (case3). T_1_ FSPGR (pre-contrast) shows a hypo-iso intensity signal mass (*arrow*). **a** STIR shows an iso-hyper intensity signal mass (0.8 cm, *arrow*) without adjacent duct expansion. **b** ADC map shows ADC = 1.30 × 10^−3^ mm^2^/s (b = 1000s/mm^2^). c TIC rapid increase (initial phases) and washout type (delayed phases). **d** MRS shows absent Cho peak at 3.2 ppm. **e** T1 FSPGR (contrast) shows an irregular, circumscribed mass with heterogeneous enhancement (*white arrow*) **(f)**

**Fig. 4:**
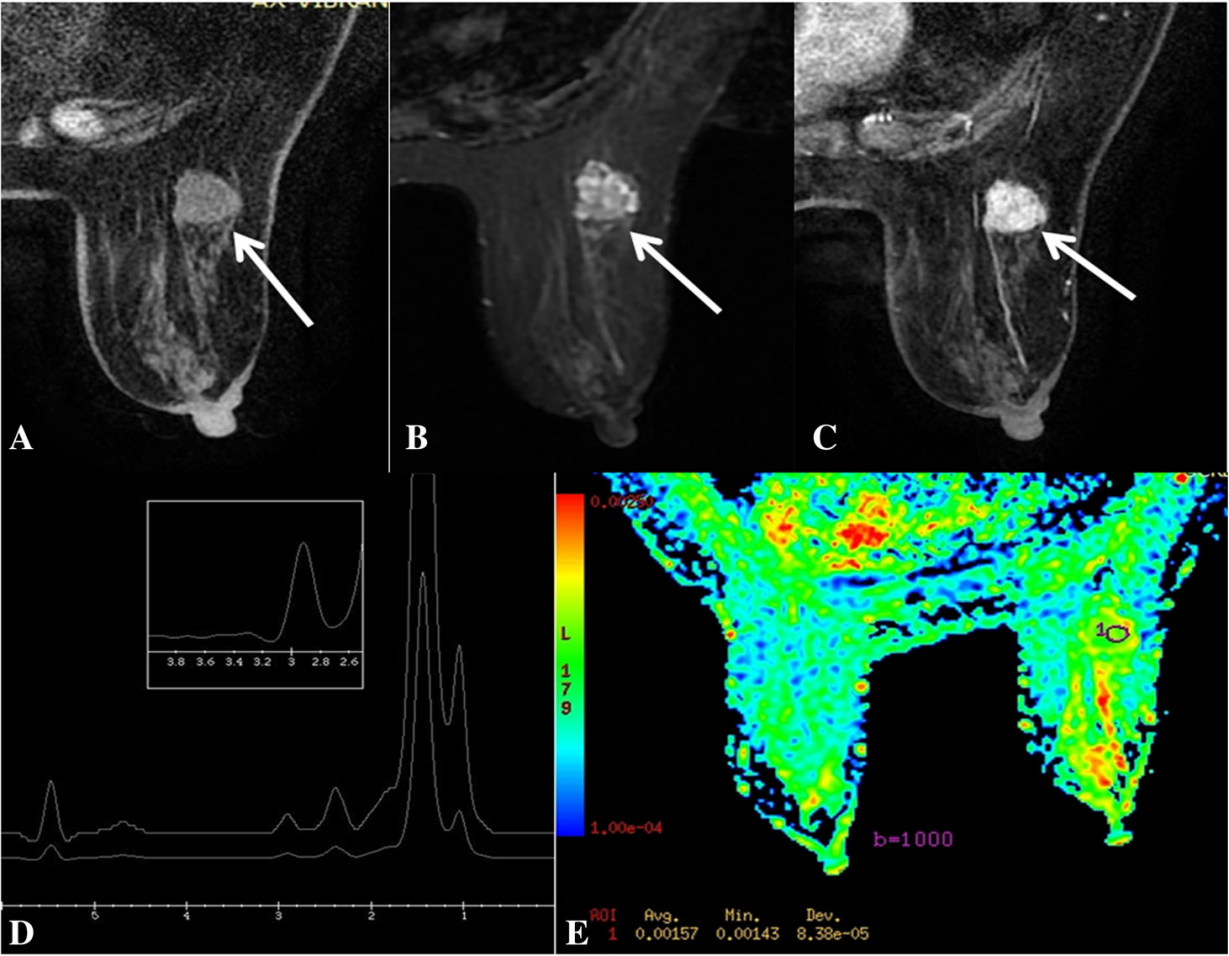
Right Breast SPC (case2). T_1_ FSPGR (pre-contrast) shows a hypo-iso intensity signal mass (*white arrow*). **a** STIR shows an iso-hyper intensity signal mass (2 cm, *white arrow*). **b** T1 FSPGR (contrast) shows an oval, circumscribed mass with heterogenous enhancement (*white arrow*). **c** ADC map shows ADC = 1.57 × 10^−3^ mm^2^/s (b = 1000s/mm^2^). **d** MRS shows absent Cho peak at 3.2 ppm. **e**

**Fig. 5:**
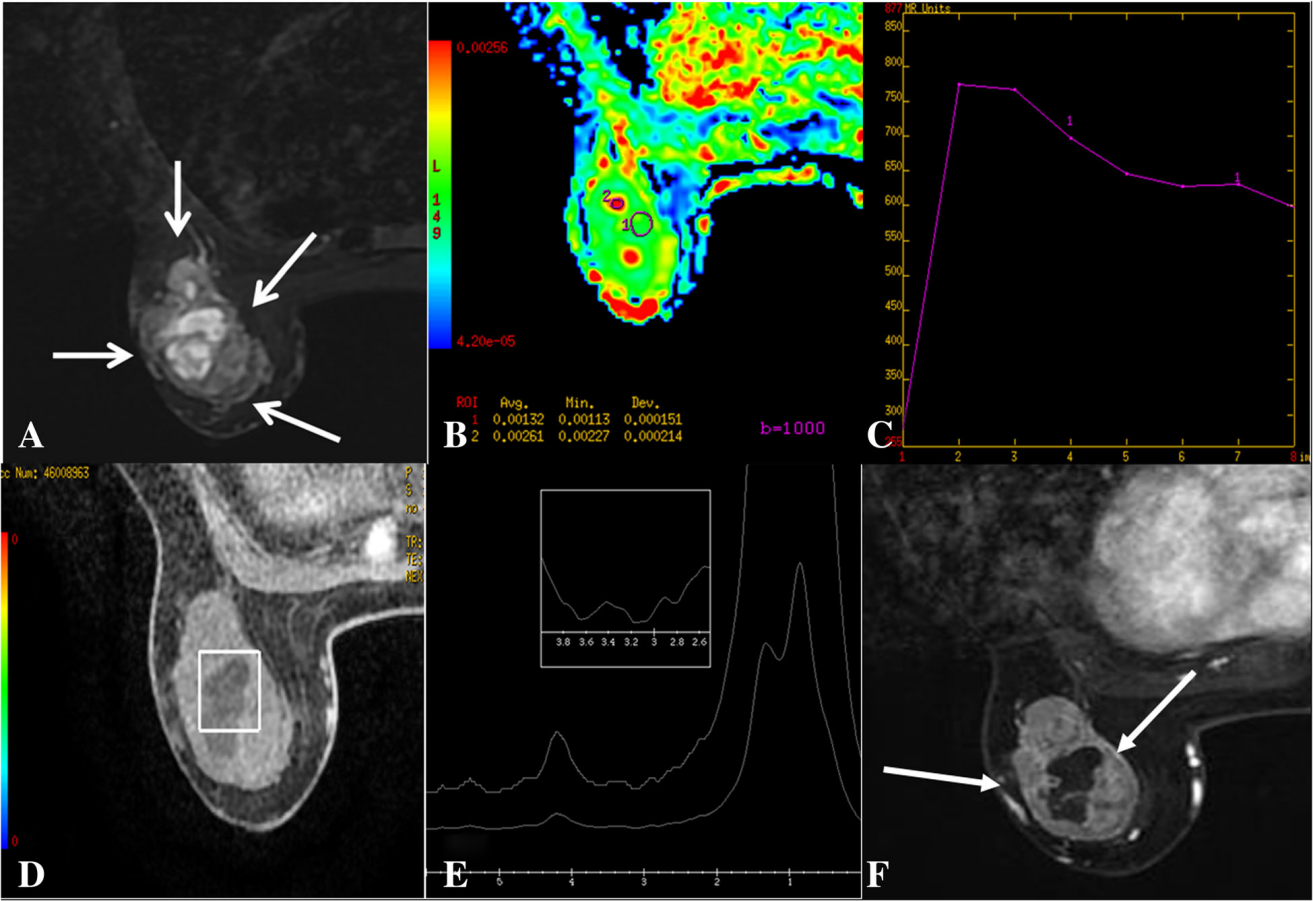
Left Breast SPC (case4). STIR shows an iso-hyper intensity signal mass (3.2 cm, *white arrow*). **a** ADC map shows ADC = 1.96 × 10^−3^ mm^2^/s (b = 1000s/mm^2^). **b** TIC rapid increase (initial phases) and washout type (delayed phases). **c** MRS shows absent Cho peak at 3.2 ppm. **d e** T1 FSPGR (contrast) shows an irregular, circumscribed mass with heterogeneous enhancement (*white arrow*) **(f)**

The maximum lesion sizes ranged from 0.8 cm to 3.2 cm among 4 patients. Features of the lesions extracted from conventional MRI are listed in Table 3. All lesions showed hypo-iso signal intensities on T_1_ FSPGR sequence (Fig. 2a, 3a, 4a), and iso-hyper signal intensities on T_2_WI and STIR sequence (Fig. 2b, 3b, 4b, 5a). On DCE-MRI, all lesions showed mass enhancement with oval (cases 1 and 2, Fig. 2c, 4c) and irregular (cases 3 and 4, Fig. 3f, 5f) shapes, and margins were circumscribed (Fig. 2c, 4c). Furthermore, internal enhancement was homogeneous in cases 1 (Fig. 2c) and heterogeneous in cases 2,3 and 4 (Fig. 4c). TIC showed rapid initial enhancement (90s) followed by plateau delayed enhancement in cases 1 and 2 (Fig. 2e), while rapid initial enhancement with washout delayed enhancement was observed in cases 3 and 4 (Figs. 3d, 5c).

**Table 3:** Conventional MRI findings of SPCs

case	Maximumlesion size(cm)	T_2_WI/STIR	T_1_ FSPGR-c	Mass enhancement		
				Shape	Margin	Internal enhancement
1	0.8	Iso-hyper	hypo-Iso	oval	circumscribed	homogeneous
2	2	Iso-hyper	hypo-Iso	oval	circumscribed	heterogeneous
3	0.8	Iso-hyper	hypo-Iso	irregular	circumscribed	heterogeneous
4	3.2	Iso-hyper	hypo-Iso	irregular	circumscribed	heterogeneous

All lesions on DWI (b = 1000s/mm^2^) were slightly hyperintense with ADC values ranging from 1.3 × 10^−3^ mm^2^/s to 1.9 × 10^−3^ mm^2^/s (Fig. 2d, 3c, 4d, 5b). Cho peak was absent at 3.2 ppm in all cases (Fig. 3e, 4e, 5d and e).

## Discussion

In our study, all lesions showed iso-hypo signal intensities on T_1_ FSPGR, and iso-hyper signal intensities on T_2_WI or STIR sequences, which included hemorrhagic or mucus components microscopically regardless of lesion size. To date, only one report by Yoshimura et al. [9] pointed out diffuse nodules surrounding the tumors in bilateral SPCs based on post-contrast T_1_ MRI.

For DCE-MRI, mass enhancement was characterized with either oval or irregular shapes, and all lesion margins were circumscribed. Furthermore, internal enhancement was homogeneous or heterogeneous. In the TIC assessment, signal intensity increased rapidly in the initial contrast phases of all lesions, which is in accordance with the TIC characteristics of most breast cancers (rapid initial enhancement) [5, 6, 11]. Meanwhile, persistent or washout type of TIC appeared in delayed contrast phases of all four cases. All SPCs features observed from DCE-MRI are in line with findings in other papillary tumors [4–7, 12, 13].

DWI is widely used to differentiate benign lesions from malignant lesions based on ADC values [4, 14, 15]. Typically, if ADC value is higher than the threshold of 1.2 × 10^−3^ mm^2^/s, the lesion is considered to be benign based on reports in the literature [11, 14]. However there are many exceptions because of the heterogeneity of tumor nature [15, 16]. In this study, ADC values of all SPC lesions were found in the range of 1.3–1.9 × 10^−3^ mm^2^/s, which is higher than the threshold. We speculate that high ADC value of SPC is due to the cystic or mucus components in the cell structures of SPC. It has been demonstrated that ADC values in mucinous cancer are higher than the threshold due to relatively free motion of water molecules in the mucin pool [16]. To further investigate whether high ADC value is a characteristic of SPC, a study including more SPC cases is underway in our institution.

The MRS results presented in this paper are not only the results of the first MRS study for SPC but also for breast papillary tumors. MRS has frequently been used to help differentiate benign breast lesions from malignant breast lesions based on the absence of signals from choline-containing compounds (Cho) [10]. Cho is a measure of increased cellular turnover and thus is generally elevated in tumors, particularly for high grade tumors. Previous studies have demonstrated that Cho signal could be low or even absent for low-grade breast tumors [10, 17, 18]. In this study, Cho peak was absent in all four cases, which suggests that the concentration of choline-containing compounds in these SPCs may be too low to be detected. The absence of Cho signal could be due to the low-grade nature of SPC in situ which grow from intervening stromal cells [18, 19]. The combination of the absence of Cho peak with a high ADC value could be a useful index to distinguish SPC in situ from other types of invasive breast carcinomas and worth further investigation with more SPC cases. Therefore, a careful study including investigating ADC values and Cho peak presentation for SPC is underway in our institution.

In our study, all patients were postmenopausal women. This finding is in accordance with previous studies which revealed that SPC primarily affects elder women [20, 21]. All lesions in our study were unilateral with no lymphadenopathy, in accordance with the majority of reports [6, 9]. Lesion size varied from 0.8 cm to 3.2 cm, which is in the range of current literature reports (1 cm to 15 cm) [6, 9, 20, 21]. However, our patients with bloody nipple discharge and accompanying dilated ducts all had lesions that were far away from the nipple. These findings differ from previous reports that SPC lesions always arise in the central (retro-areolar) area of the breast with no specific clinical features at presentation [6, 9, 22].

## Conclusion

In conclusion, SPC MRI is characterized by heterogeneous signal intensity within the lesion, mass enhancement with circumscribed margins, either oval or irregular shapes, and the presence of a rapid enhancement in initial contrast phases. Moreover, high ADC values and the absence of Cho peak may provide valuable information for distinguishing SPC from other invasive breast carcinomas.

## Abbreviations

ADC: Apparent Diffusion Coefficient
DCE: Dynamic Contrast Enhanced
DWI: Diffusion Weighted Imaging
FSPGR: Spoiled Gradient Echo Fat Saturation
IHC: Immunohistochemistry
MRI: Magnetic Resonance Imaging
MRS: Magnetic Resonance Imaging
SPC: Solid Papillary Carcinoma
TIC: Time of Intensity Curve
